# Pro-197-Ser Mutation in *ALS* and High-Level GST Activities: Multiple Resistance to ALS and ACCase Inhibitors in *Beckmannia syzigachne*

**DOI:** 10.3389/fpls.2020.572610

**Published:** 2020-09-30

**Authors:** Jingjing Wang, Jingchao Chen, Xiangju Li, Dan Li, Zheng Li, Hailan Cui

**Affiliations:** Institute of Plant Protection, Chinese Academy of Agricultural Sciences, Beijing, China

**Keywords:** herbicide resistance, weeds, metabolism, RNA-seq, overexpression

## Abstract

American sloughgrass (*Beckmannia syzigachne* Steud.) is one of the most troublesome weeds infesting wheat and canola fields in China. Some biotypes cannot be controlled, either by acetolactate synthase (ALS) or acetyl coenzyme A carboxylase (ACCase) inhibitors, which are the main herbicides for controlling this weed. However, very few studies have investigated multiple resistance mechanism in *B. syzigachne*. In this study, a *B. syzigachne* biotype with a high resistance to ALS inhibitors we have reported was also showed relatively lower resistance to ACCase inhibitors, with a resistance index around 7. RNA-seq analysis was used to investigate the factors responsible for multiple resistance, and 60,108 unigenes were assembled by *de novo* transcriptome assembly and then annotated across eight databases. A Pro-197-Ser mutation was identified in the *ALS* gene by SNPs analysis and validated by PCR, while no mutation was identified in the *ACCase* gene. Nineteen candidate metabolic genes were screened and their overexpression was confirmed by qPCR. The expression of *GST-T3* and *GST-U6* in resistant plants ranged from 7.5- to 109.4-folds than that in susceptible ones at different times after two kinds of herbicide treatment. In addition, GST activities in resistant plants were 3.0–5.0 times higher than that in susceptible plants. Other novel resistance factors also showed high correlation with multiple resistance which included four genes encoding disease resistance proteins, a transcription factor (*MYC3*), and one gene conferring blight resistance. In this research, a *B. syzigachne* biotype was confirmed to have evolved multiple resistance to ACCase and ALS inhibitors. The Pro-197-Ser mutation in *ALS* gene and high-level GST activities were confirmed responsible for the multiple resistance. Characterized disease-resistance proteins, transcription factor, and blight-resistance proteins may play an essential role in these multiple herbicide resistance.

## Introduction

American sloughgrass (*Beckmannia syzigachne* Steud.) is a troublesome diploid weed (2n = 14), which threatens many crop varieties in China such as wheat (*Triticum aestivum* L.) and oilseed rape [*Brassica rapa* L. subsp. Oleifera (DC.) Metzg.] (Rao et al., 2008; Li et al., 2015; Du et al., 2016; Li et al., 2017). Inhibitors of acetyl coenzyme A carboxylase (ACCase; EC 6.4.1.2) and acetolactate synthase (ALS; EC 4.1.3.18) are the main herbicides that are used to control the *B. syzigachne* in China. However, after many years of selection pressure, this weed evolved cross- or multiple-resistance to these two mechanisms of herbicides (Li et al., 2013; Li et al., 2015; Li et al., 2017). Resistant *B. syzigachne* can survive under high doses of these two kinds of herbicides *via* a variety of mechanisms. We sought to investigate resistance mechanisms useful for its control.

Based on the confirmed resistance against the ACCase-inhibiting and ALS-inhibiting herbicides in weeds, two kinds of resistance mechanisms can be summarized; these are target-site resistance (TSR) and non-target-site resistance (NTSR) (Délye, 2005). TSR is caused by amino acid substitutions in the conserved regions or due to differences in the expression of target enzyme genes, and has been well described (Délye, 2005; Powles and Yu, 2010). A mutation in the carboxyl-transferase (CT) domain of *ACCase* causes weed resistance to ACCase-inhibitors and confers distinct cross-resistance patterns (Powles and Yu, 2010; Scarabel et al., 2011; Beckie and Tardif, 2012; Kaundun, 2014). With respect to *ALS*, an amino acid substitution is commonly found at eight positions, and the different mutations also result in different levels of resistance against this kind herbicide (Yu and Powles, 2014b; Heap, 2019). NTSR is complicated and commonly results due to the overexpression of a set of genes that lead to reduced herbicide absorption, translocation, or increased metabolism and sequestration (Délye, 2013). To date, many well-established gene families are known to be involved in NTSR, such as the cytochrome P450 monooxygenase (P450s), and glutathione S-transferase (GSTs) families (Yuan et al., 2007). Many genes of these families are confirmed to have conferred resistance in herbicide-resistant weeds (Iwakami et al., 2014; Saika et al., 2014; Iwakami et al., 2019).

It has been reported that the mechanism governing the resistance of *B. syzigachne* to ACCase-inhibiting herbicides mainly involves TSR, in which amino acid substitutions have been identified at six positions (1781, 1999, 2027, 2041, 2078, and 2096) in resistant *B. syzigachne* (Du et al., 2016; Liu et al., 2019). Only the mutation involving Pro197Ser was found in the *B. syzigachne* population resistant to ALS-inhibiting herbicides (Li et al., 2015; Wang et al., 2018). For NTSR, metabolic genes related to resistance have been reported in fenoxaprop-P-ethyl-resistant *B. syzigachne* (Pan et al., 2016a; Pan et al., 2016b). However, to our knowledge, no studies have reported the involvement of genes in the NTSR mechanisms in *B. syzigachne* with ALS resistance, though the NTSR mechanisms have been confirmed (Wang et al., 2018; Wang et al., 2019; Bai et al., 2020). Furthermore, resistance genes relative to multiple resistance to ACCase and ALS-inhibiting herbicide have not been reported. Recently, we found a *B. syzigachne* biotype with ALS-inhibiting herbicide resistance which confirmed in our previous study was also hardly controlled by the ACCase inhibitors (Wang et al., 2018).

Hence, this research aimed to (1) determine the multiple resistance levels of *B. syzigachne* to ALS- and ACCase-inhibitors, (2) explore the multiple resistance factors using next generation sequencing methods, (3) confirm the Single Nucleotide Polymorphisms (SNPs) or the expression for the candidate genes, and (4) determine the enzyme activity which relative to the multiple resistance.

## Materials and Methods

### Plant Material and Whole Plant Assays

Resistant (R) *B. syzigachne* biotype were collected from Wuxi city (WC1148) and found evolved high-level resistance to ALS inhibitors in our previous study (Wang et al., 2018). A susceptible population (S) (WC1004), with no history of herbicide use was collected from near the R biotype. The plants of these two populations were cultured using the methods described in a previous study and thinned to 10 individuals per pot (Wang et al., 2018). Three kinds of ACCase inhibitor herbicide were selected for resistance assays (**Table S1**). The herbicides were applied when the seedlings reached the 3-leaf stage using a spray chamber with a moving TeeJet^®^ XR8002 flat fan nozzle under a pressure of 0.275 MPa (Wang et al., 2018). Dry weights of the shoots (dried at 80°C for 48 h) in each pot were measured 21 days after treatment (DAT). The experiment of whole plant assay was repeated twice in a completely randomized design that had three biological replicates (three pots per treatment).

### Sample Collection and RNA Extract for RNA-Seq

The plant culture conditions for the R and S populations were the same as in previous reports (Wang et al., 2018). The experimental design included three different treatment for R and S: without herbicide treatment control (CK), mesosulfuron-methyl treatment (M), and fenoxaprop-P-ethyl treatment (F). When the individuals reached the 3–5 leaf-stage, each biotype was treated with 70 g a.i. ha^-1^ of fenoxaprop-P-ethyl and the 20 g a.i. ha^-1^ mesosulfuron-methyl, respectively. After 24 h, the leaf tissues of three biological replicates were random collected, and also the samples without herbicide treatment were collected as controls with three individuals for each biotype. All the 18 samples were snap frozen in liquid nitrogen and stored at -80°C. Total RNA was extracted using the RNAprep Pure Plan Kit (Tiangen Biotech Beijing CO., LTD, Beijing, China). The degradation and contamination were checked by 1.0% agarose gel electrophoresis. RNA purity and concentration were determined using the NanoDrop-1000 spectrophotometer (NanoDrop Technologies, Wilmington, DE, USA). The RNA integrity was assessed by the Agilent Bio analyzer 2,100 system (Agilent Technologies, Palo Alto, USA) (Chen et al., 2017a).

### Construction of cDNA Libraries, Sequencing, and Bioinformatics Analysis

The cDNA library construction was performed by a common method using the 18 detected RNA samples by Allgwegene Health Co. (Beijing, China) (Chen et al., 2017a). Paired-end reads (125 bp) were determined using an Illumina HiSeq™ 4000 (Illumina Inc., San Diego, CA, USA) platform, and clean reads were obtained from which raw reads with low quality were removed. Subsequently, *de novo* transcriptome assembly was carried out using the Trinity platform and the longest transcript of each locus was termed a “unigene” for subsequent annotation (Grabherr et al., 2011). Eight databases were selected for the annotation of the unigenes (**Table S2**). The software BLASTX (E-value < 10^-5^), BLAST2GO, and Blastall were used to predict and classify the different unigenes, as in previous studies (Tatusov et al., 2001; Ana et al., 2005; Clark et al., 2007).

### SNPs Analysis for Target Genes and PCR Validation

For SNP calling, picard tools was used to map the paired-reads from each sample to the reference genome sequence of *Brachypodium distachyon* [L.] Beauv. The software GATK2 was used for SNP discovery and the detection stringency conditions include at least five reads calling the variant and >30 mapping quality score (Mckenna et al., 2010). To confirm the SNPs results for *ALS* and *ACCase* gene, the PCR method was selected to investigate the mutations in these two genes. DNA of the leaf tissues was exacted (15 individuals both for R and S populations) using the Hi-DNAsecure Plant Kit (DP350) by following the manufacturer’s instructions (Tiangen Biotech Beijing CO., Ltd., Beijing, China). Two pairs of PCR primers were design to clone *ALS* (1F- 5’CGCCTTACCCAAACCTACT3’, 1R- 5’ATGCGGCTGCTTGTTCTT3’; 2F- 5’ATCCCACCACAATATGCTATCC3’, 2R- 5’TCACAGTTGACCACACTTC3’); the amplification products of these two pairs of primers were 1035 and 766 bp in size, respectively, which encompass all of the reported mutation sites. One pair of primers was designed (F-5’AAACTCTGGTGCTCGGATTG3’; R-5’TAGGCTTCCATTTGCTCCC3’) to clone *ACCase* containing the CT region, and the PCR products were 1308 bp in size. The PCR mixture components and amplification conditions were in accordance with those reported in a previous study (Chen et al., 2017b), and the annealing temperature for all the primer pairs was 58°C. After confirming fragment amplification using 1% agarose gels prepared in 1× TAE, followed by staining with ethidium bromide, the PCR products were sequenced by Tsingke (Tsingke Biological Technology, Beijing Co., Ltd., Beijing, China), and the *ALS* and *ACCase* sequences of all the populations were aligned using the software DNAMAN (Lynnon BioSoft, San Ramon, CA, USA). The experiment of PCR was done twice and had 15 biological replicates both for R and S biotypes.

### Analysis of Differentially Expressed Genes

Expression analysis was performed for the selected 18 samples, which were same as described above, and the samples were divided into different groups. The samples of the R and S biotypes without herbicide treatment were named CK1148 and CK1004, respectively. The sample groups treated with mesosulfuron-methyl for the R and S biotypes were named M1148 and M1004, respectively. Similarly, the samples groups treated with fenoxaprop-P-ethyl for the R and S populations were named F1148 and F1004, respectively. The cDNA library constructs and the clean reads detected for the 18 samples were the same as described above. The clean reads for the 18 libraries were mapped back onto the reference transcriptome, which was assembled in the above part by Bowtie 2 v.2.2.3 (Li and Dewey, 2011). The expression of each gene between sample pairs (CK1148 vs. CK1004, F1148 vs. CK1148, M1148 vs. CK1148, F1004 vs. CK1004, M1004 vs. CK1004, F1148 vs. F1004, and M1148 vs. M1004) were analyzed both by the numbers of reads and fragments aligned per thousand bases per million reads (FPKM), which was conducted using DESeq 2 v.1.4.5 (Audic and Claverie, 1997; Love et al., 2014). The false discovery rate is considered to be a standard to confirm the threshold of *P* values in multiple tests and analyses (Benjamini and Yekutieli, 2001).

### Candidate Resistance Gene Selection and qPCR Validation

Candidate resistance genes were selected considering the reported NTSR genes include metabolism and signaling functions genes, the statistical significance (*q* < 0.05), and magnitude of differences in expression between the above treatment groups (|log_2_ (fold change)| ≥ 1). quantitative Real-time PCR (qPCR) was performed to confirm the accuracy of their expression. The best reference gene was selected from among capsine phosphatase (*CAP*), glyceraldehyde 3-phosphate dehydrogenase (*CADP*), and ubiquitin (*UBQ*) by the software BestKeeper (Pfaffl et al., 2004). The plant culture method, herbicide treatment for the individuals, and sample selection and storage were the same as described above. Total RNA was exacted, and first strand cDNA synthesis was performed (Tiangen Biotech Co., Ltd., Beijing, China) for all samples, and then primers were designed for all the genes (reference genes and the candidate resistance genes) using the software Oligo 7.0 (**Table S3**). The qPCR reaction was performed in 25 µL reaction volumes on an ABI 7500 PCR instrument under common conditions (Chen et al., 2017a). Relative expression of the selected genes was quantified using the 2^-ΔΔ^Ct method (Bustin et al., 2009). For the qPCR, three biological replicates were performed for each treatment, and three technical replicates was done for each sample.

### Expression and Enzyme Activity Validation of GSTs

The plant culture for the R and S biotypes, and the herbicide treatment were same as described above. At 0, 24, 48, and 72 h, leaf samples for each treatment (at least three replicates) were collected and stored at -80°C. Total RNA was exacted and first strand cDNA synthesis was performed using the methods described above for all samples. The expression levels of two GSTs-related genes (*GST-T3* and *GST-U6*) were detected using the qPCR method for all the samples, respectively, and the GST activity for the samples were also detected using a Glutathione S-transferase Microplate Assay Kit (CAK 1047) following the manufacturer’s instructions (Cohesion Biosciences Co., Ltd., Suzhou, China). Each sample (0.1 g leaf tissues) was assayed at 340 nm using a Tecan Infinite 200 Pro plate reader (Tecan Group Ltd., Männedorf, Switzerland). The results were expressed as U g^-1^, and one unit of GST activity was defined as the enzyme generating 1 μmol of glutathione and 1-chloro-2, 4-dinitrobenzene conjugate in the reaction time. Both the gene expression and GST activity assay were repeated twice and at least three biological replicates were performed for each treatment.

### Statistical Analysis

The values of herbicide doses required to reduce 50% of the plant growth (GR_50_) were estimated through nonlinear regression with a log-logistic model:

$$Y=y^{0}+\frac{a}{1+{\left(\frac{X}{X_{0}}\right)}^{b}}$$

using Sigma Plot 12.0 (Systat software, San Jose, CA, USA) (Seefeldt et al., 1995). In this model, *b* is the slope of the curve, *y_0_* is the lower limit, *a* is the difference between the upper limit and the lower limit, and *X_0_* is the GR_50_. Resistance levels were determined by calculating the ratio of the GR_50_ of the resistant biotype to that of the susceptible biotype, and the results are expressed as resistance index (RI). The difference of gene expression and GST activity between R and S biotypes were analyzed by the Student’s t-test, which were performed with SPSS 13.0 (SPSS, Chicago, USA).

## Results

### Resistance Levels to ALS- and ACCase-Inhibiting Herbicides

The sensitivity of the R (WC1148) and S (WC1004) biotypes in response to four different ACCase inhibitors (fenoxaprop-P-ethyl, clodinafop-propargyl, clethodim, and pinoxaden) was calculated. The GR_50_ values of the S biotype WC1004 were 42.4, 12.1, 11.4, and 11.8 g a. i. ha^-1^, respectively. For the R biotype WC1148, the GR_50_ values were 87.0, 39.4, 84.5, and 89.2 g a. i. ha^-1^, respectively. Compared with those of the sensitive biotypes, the RI of WC1148 were 2.1, 3.3, 7.4 and 7.5, respectively (**Table 1**). These results indicated that the ALS inhibitors resistant biotype WC1148 evolved multiple resistance to the ACCase inhibitors.

**Table 1:** GR_50_ values and resistance index of *B. syzigachne* to ACCase inhibitors in the R and S biotypes.

Classification of herbicide^a^		Herbicides	Biotypes^b^	GR_50_ ± SE (g a.i.ha^-1^)	P-value	Resistanceindex (RI)^c^
ACCase inhibitors	APPs	Fenoxaprop-P-ethyl	S	42.4 ± 9.8	<0.001	—
			R	87.0 ± 24.8	<0.001	2.1
		Clodinafop-propargyl	S	12.1 ± 9.2	<0.001	—
			R	39.4 ± 7.8	<0.001	3.3
	CHDs	Clethodim	S	11.4 ± 5.7	<0.001	—
			R	84.5 ± 45.5	<0.001	7.4
	PPZs	Pinoxaden	S	11.8 ± 3.6	<0.001	—
			R	89.2 ± 65.9	<0.001	7.5

a APPs, Aryloxyphenoxypropionates; CHDs, Cyclohexanediones; PPZs, Phenylpyrazoline;

b S: susceptible biotype; R: resistant biotype; GR_50_: herbicide doses required to inhibit dry weight by 50% compared to untreated controls;

c RI, resistance index, and determined by dividing the GR_50_ of the R by that of S biotype.

### RNA-Seq and *De Novo* Assembly, Gene Annotation, and Functional Classification

The OD_260_/OD_280_ values (around 2.0) and the RNA integrity number (RIN) values (>6.0) indicated that the RNA integrity of the samples was suitable for the requirements of subsequent experiments. The cDNA library of 18 RNA samples were analyzed: 449,583,465 raw reads, and 436,188,739 clean reads which ranged from 22,724,569 to 24,516,824 per sample were generated, respectively (**Table S1**). We obtained 118,111 unigenes that ranged from 201 to 21,671 bp in size and with an N50 of 1,328 bp.

More than half (50.89%) of the total 118,111 unigenes were successfully annotated in at least one database, and 8,197 unigenes were annotated in all of the database (**Table S1**). 35,616 unigenes were assigned into 42 functional categories by the Gene Ontology (GO) database and including 20 for “biological process”, 12 for “cellular component”, and 10 for “molecular function” (**Table S4**). In total, 25,598 unigenes were categorized into 26 Clusters of Orthologous Groups of proteins (KOG) classifications (**Table S5**). The 7,893 assembled sequences were mapped to the reference canonical pathway in Kyoto Encyclopedia of Genes and Genomes (KEGG) (**Table S6**). The 48,600 unigenes were assigned putative annotations by the NCBI non-redundant protein sequences (NR), 39,869 unigenes by NCBI non-redundant nucleotide sequences (NT), 31,291 unigenes by a manually annotated and reviewed protein sequence database (SwissProt), and 39,310 unigenes in the Protein family (Pfam) database.

### SNPs Analysis and PCR Validation for *ALS* and *ACCase* Gene

**Figure 1:**
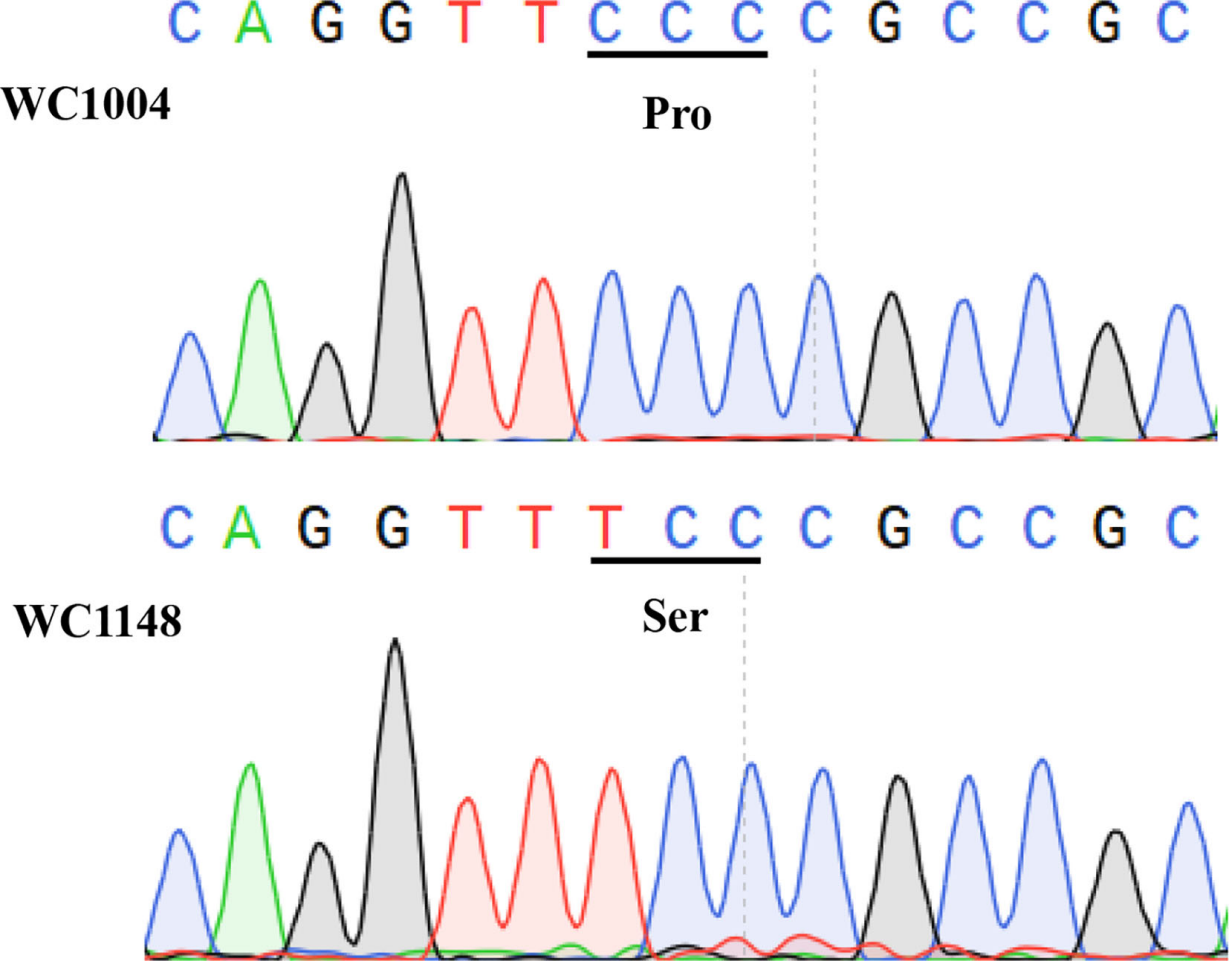
Sequences result for *ALS* genes in WC1148 and WC1004 biotypes, respectively. A Pro-107-Ser mutation in the *ALS* gene evolved in the resistant biotype WC1148, and DNA sequencing chromatograms show the homozygous mutation.

Two SNPs were detected for the *ALS* gene (c38811_g1) using the RNA-seq analysis, while, no SNPs was found for the *ACCase* gene (**Table S7**). For the *ALS* gene, one SNP was only found in the resistant individuals (C to T at position 629) which causing the amino acid Pro change to Ser (**Table S7**). While, another SNP (G to A at position 820) was only found in the susceptible individuals but without causing any amino acid change. To confirm the results of the SNPs assay, parts of the *ALS* and *ACCase* genes which contain all the common mutation sites of these two kinds of inhibitors in *B. syzigachne* were cloned from the R and S populations. Comparing the conserved regions of the *ALS* gene for the S and R biotypes, it was found that 197 sites of *ALS* were mutated from proline CCC (Pro) to serine TCC (Ser), and no other mutation sites were found in any of the 15 individuals (**Figure 1**). By comparing the *ACCase* CT region of the R and S biotypes, no mutation was found in the CT region of the *ACCase* gene. The results of the chromatograms show that the *ALS* gene in the R biotypes was a single peak at the mutation site, indicating a homozygous mutation (**Figure 1**), and the detected 15 individuals of R biotypes showed the same mutation type.

### Analysis of Differentially Expressed Genes

**Figure 2:**
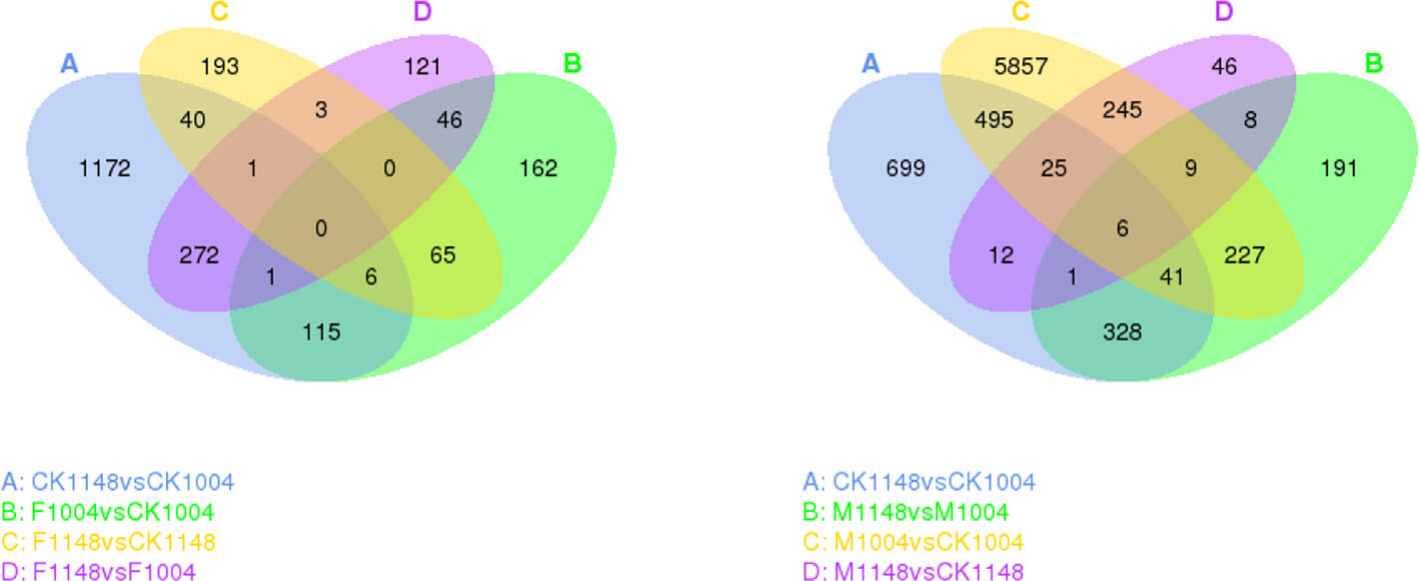
Venn diagram showing differential gene expression between the different group sets for the resistant biotype WC1148 and susceptible biotype WC1004. The circle represents the comparisons, the sum of the numbers in each circle represents the genes showing differential expression, and the overlapping part of the circle represents the number of the same genes between different comparisons.

Seven different comparison combinations were designed as the biotype WC1148 showed multiple resistance to ALS and ACCase inhibitor herbicides (**Figure 2**). Differentially expressed genes between these groups (|log_2_ (fold change)| ≥ 1 and q < 0.05) were selected as candidate resistance genes. The number of up- and down-regulated genes in CK1148 vs CK1004 combination was 700 and 907, respectively. For the group set of M1148 vs CK1148, and F1148 vs CK1148, the number of up- and down-regulated genes was no more than 300. After herbicide treatment, differentially expressed genes between the R and S biotypes (M1148 vs. M1004 and F1148 vs. F1004) were more than 400. The number of up- and down-regulated genes in the M1004 vs. CK1004 combination was 2,720 and 4,185, respectively. However, the number of up- and down-regulated genes in the F1004 vs. CK1004 combination was 162 and 233, respectively (**Table 2**).

**Table 2:** Number of differentially expressed genes among different groups.

Treatment groups	Differentially expressed gene	Up-regulated	Down-regulated
CK1148 vs. CK1004	1607	700	907
M1148 vs. CK1148	352	274	78
F1148 vs. CK1148	308	115	193
M1004 vs. CK1004	6905	2720	4185
F1004 vs. CK1004	395	162	233
M1148 vs. M1004	811	393	418
F1148 vs. F1004	444	203	241

CK: untreated herbicides; M: mesosulfurn-methyl treatment; F: fenoxaprop-p-ethyl treatment.

### Selection of Candidate Non-Target Site Resistance Genes

We analyzed and selected factors related to NTSR based on the reported mechanism in weeds (Délye, 2013; Yu and Powles, 2014a; Pan et al., 2016a). The reported genes relative to the metabolic enzyme families in *B. syzigachne* were considered which include *CytP450*, *GST*, *ABC* transporter family, and esterase gene families (Pan et al., 2016a). Other genes up-regulated in all the comparative groups were considered, especially for up-regulated genes in CK1148 vs. CK1004. After preliminary analysis of the different expression of the genes between different treatment groups, 19 candidate genes were related to resistance. Among these candidate genes, common metabolic genes were identified, which included three genes annotated as GSTs, one gene being an ABC transporter, two protein kinase genes, one annotation as UDP-glycosyl transferase, and two for oxidases. In addition, certain novel resistant factors were found, which included two immune proteins, five disease resistance proteins, two termed ‘other’ transferase, and one transcription factor (**Table 3**).

**Table 3:** Candidate genes related to mesosulfuron-methyl and fenoxaprop-p-ethyl metabolism in *B. syzigachne*.

Gene ID	Gene annotation	Fold-change(F1148 vs F1004 or M1148 vs M1004)	Padj
c59327_g1	GST-T3	8.68	2.22 × 10^-20^
c9871_g1	GST-U6	4.82	2.62 × 10^-20^
c81158_g1	GST-U6	5.46	2.05 × 10^-3^
c61256_g2	ABC G family member 15	5.81	3.57 × 10^-20^
c52253_g1	UDP-glycosyltransferase 88F5	6.57	6.51 × 10^-20^
c30288_g1	LRR RLK At4g08850	8.36	1.60 × 10^-9^
c64517_g5	LRR RLK At1g56140	3.95	8.94 × 10^-3^
c61823_g3	Iron/ascorbate family oxidoreductases	6.82	9.95 × 10^-7^
c50888_g2	L-pipecolate oxidase	4.03	3.25 × 10^-3^
c61143_g4	Late blight resistance protein R1B-16	10.86	6.83 × 10^-20^
c71182_g1	Late blight resistance protein R1A-10	7.76	2.59 × 10^-4^
c41866_g1	Disease resistance protein At1g15890	10.52	7.67 × 10^-13^
c62003_g3	Disease resistance protein At1g58602	9.99	4.94 × 10^-10^
c63887_g5	Disease resistance protein RPP13	5.33	1.10 × 10^-6^
c64184_g2	Disease resistance protein RGA3	4.30	1.15 × 10^-4^
c51431_g2	Disease resistance protein RPP13	7.11	5.85 × 10^-4^
c59149_g1	Polyamine aminopropyltransferase	5.99	1.40 × 10^-10^
c45597_g1	Anthranilate N-benzoyltransferase protein 1	6.79	6.83 × 10^-4^
c96180_g1	Transcription factor MYC3	3.77	4.11 × 10^-2^

### qPCR Validation of Candidate Metabolic Resistance Gene Expression

**Figure 3:**
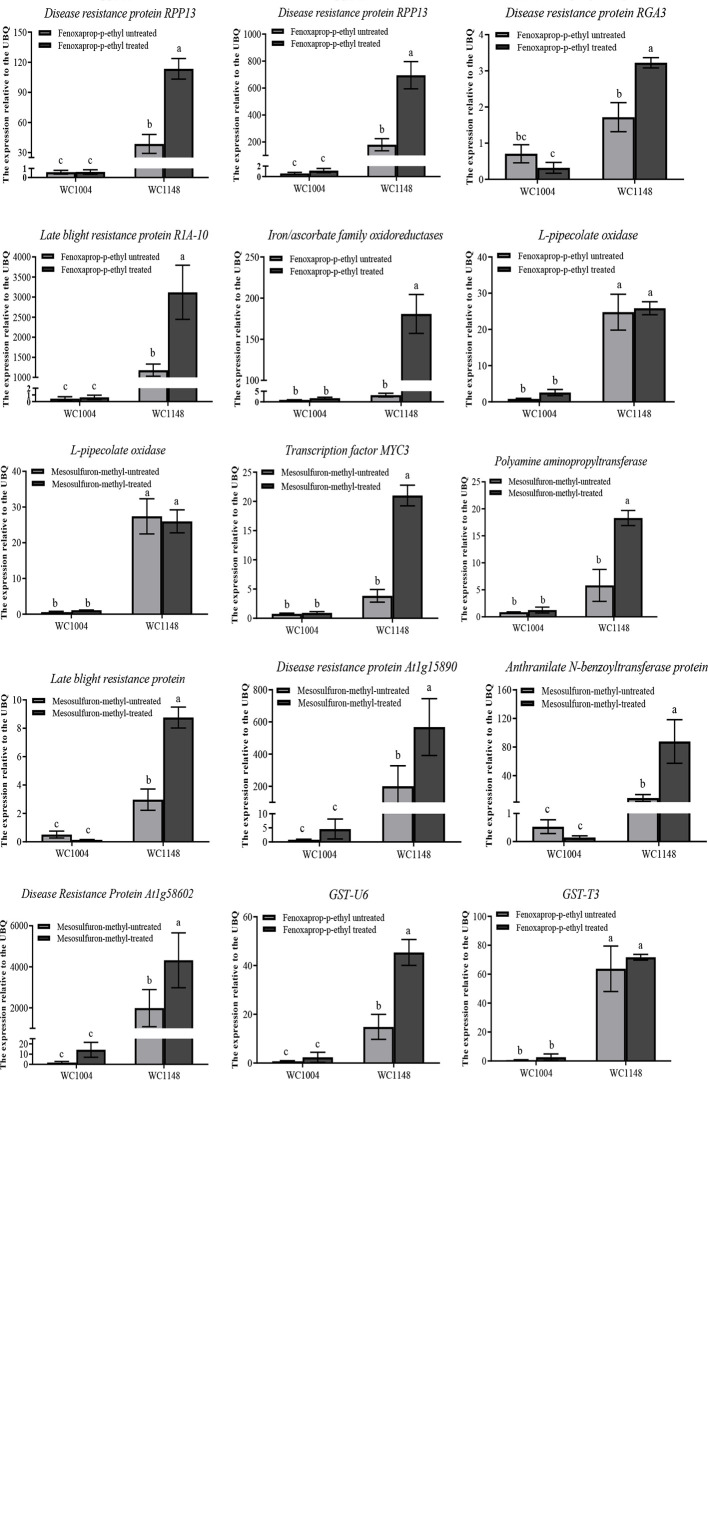
Relative expression validations for candidate resistance genes using the qPCR method. The confirmed reference gene *UBQ* was selected as an internal control gene. The resistant biotype named WC1148 and the susceptible biotype named WC1004. Three biological replicates were performed for each treatment, and three technical replicates was done for each sample. Vertical bars show the standard error of the mean for the replicates. Different lowercase characters indicate significant differences in expression between the treatments.

The expression of the selected contigs were confirmed using the qPCR method, and UBQ was the best reference gene which analyzed by Bestkeeper (**Table S8**) (Pfaffl et al., 2004). The expression levels of 13 candidate resistance genes were validated by qPCR at 0 and 24 h after treatment with 20 g a.i. ha^-1^ mesosulfuron-methyl for sensitive R and S biotypes. The results showed that in the absence of treatment with the ALS inhibitor mesosulfuron-methyl, seven genes, including *GST-U6*, *disease resistance protein At1g4809*, and *disease resistance protein At1g58062* in the R biotype, exhibited 5.8-1124.0 times higher expression than that in S (**Figure 3**). However, other genes show similar expression level between the R and S biotypes (**Figure 3**). After this herbicide treatment, all these candidate genes showed 5.9–4928.2 times higher expression levels in the R biotype than that in the S biotype.

Moreover, eight candidate resistance genes were validated by qPCR at 0 and 24 h after treatment with 70 g a.i. ha^-1^ fenoxaprop-p-ethyl. Among these genes, six showed higher expression in the R biotype both before and after herbicide treatment than that in S biotype. In particular, for the gene *disease resistance protein RPP13*, without herbicide treatment, the expression level in the R biotype was 300 times higher than in the S biotype (**Figure 3**). After herbicide treatment, the expression could reach 700 times higher.

### Expression and Enzyme Activity Validation of GSTs

**Figure 4:**
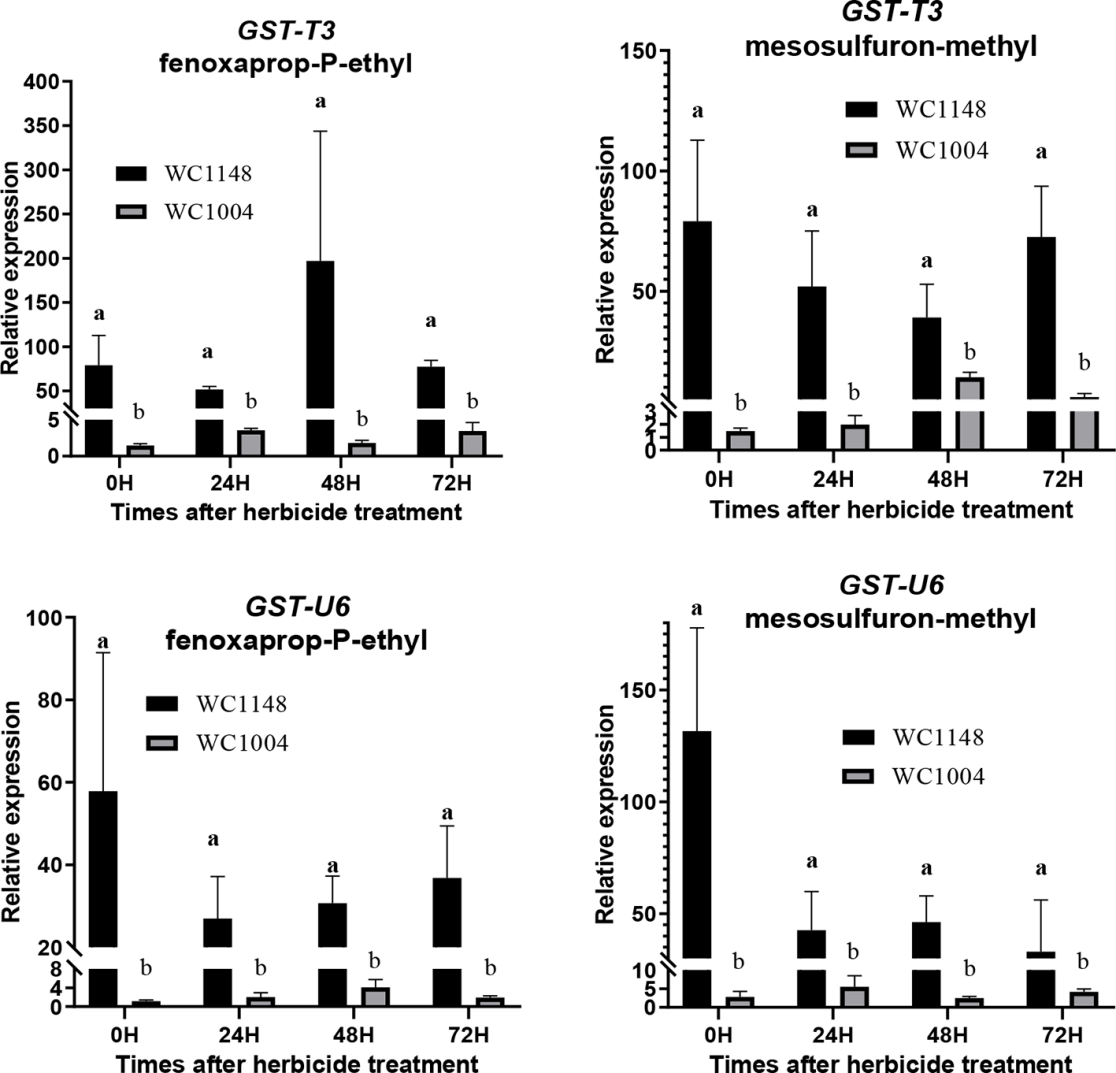
The expression of two unigenes (*GST-T3*, *GST-U6*) annotation to GSTs in the resistant (R) and susceptible (S) plants at 0, 24, 48, and 72 h after mesosulfuron-methyl (20 g a.i. ha^-1^) and fenoxaprop-P-ethyl (70 g a.i. ha^-1^) treatment, respectively. At least three biological replicates were performed for each treatment, and three technical replicates was done for each sample. Bars are means ± standard error of the mean (SEM). Different lowercase means the significant difference at each time point between R and S which analyzed by the T-test (*P <* 0.05).

The expression levels of two unigenes (*GST-T3*, *GST-U6*) annotation to GSTs were analyzed at different times after herbicide treatment. The results show that expression of these two genes in the R individuals was higher than that in the S individuals at each time point under herbicide treatment (**Figure 4**). After mesosulfuron-methyl treatment, the expression of *GST-T3* in R can reach to 53.8-folds than S, similar results (108.5-folds) were also found after fenoxaprop-P-ethyl treatment. Without herbicide treatment, the expression of *GST-U6* was 46.5 times higher than in S and show slight decreased after these two kinds herbicide treatment.

**Figure 5:**
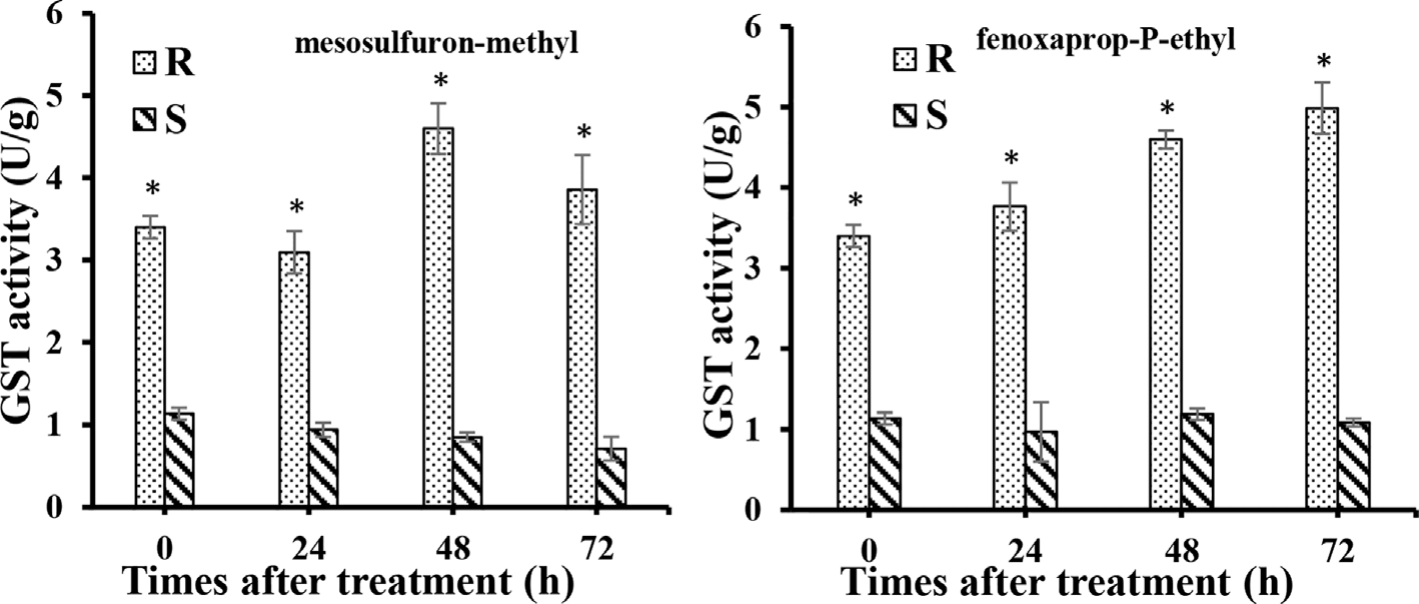
GST activities in the resistant (R) and susceptible (S) plants at 0, 24, 48, and 72 h after fenoxaprop-P-ethyl (70 g a.i. ha^-1^) and mesosulfuron-methyl (20 g a.i. ha^-1^) treatment, respectively. At least three biological replicates were performed for each treatment. Bars are means ± standard error of the mean (SEM). *Significant difference at each time point between R and S (*P <* 0.05).

To determine whether the higher expression of these genes of GSTs increase the GST active in R biotype. GST activity was investigated in both R and S biotypes at 0, 24, 48, and 72 h after mesosulfuron-methyl and fenoxaprop-P-ethyl treatment. The results show that in R plants without herbicide treatment, the GST activity was 3.4 U g^-1^, which was 3.0 times higher than that in the S plants (**Figure 5**). After mesosulfuron-methyl treatment, GST activities were slightly increased in R biotype and reached a peak at 48 h (4.6 U g^-1^), which was 5.0 times higher than that in S biotype. However, it was slightly decreased in the S biotype, which is around 0.8 U g^-1^. Similar results were also found in these two biotypes after fenoxaprop-P-ethyl treatment. The GST activities were increased in R biotype and can reach to 4.9 U g^-1^, while GST activities in the S biotype was around 1.0 U g^-1^ at all the time points. GST activities in the R plants were significantly higher than those in the S plants at all the time points.

## Discussion

### Multiple Resistance Levels to ALS and ACCase Inhibitors

In this study, we confirmed that a *B. syzigachne* biotype had evolved multiple resistance to ALS and ACCase inhibitor herbicides. In our previous research, this biotype showed higher resistance to mesosulfuron-methyl, and also exhibited higher cross-resistance to other ALS inhibitor herbicides, such as flucarbazone, imazapic, pyroxsulam, and pyribenzoxim (Wang et al., 2018). In this study, the same biotype shows relatively low resistance levels to the ACCase inhibitor herbicide, with resistance levels ranging from 2.1 to 7.5. Cross-resistance is a common phenomenon in weed species resistant to ALS and ACCase inhibitor herbicides, such as *Conyza canadensis* (Zheng et al., 2011), *Myosoton aquaticum* L. (Liu et al., 2015), and *Bromus tectorum* L. (Park and Mallory-Smith, 2004). *Beckmannia syzigachne*, biotypes with cross-resistance to ACCase and ALS inhibitor herbicides are frequently reported in China (Li et al., 2013; Li et al., 2014; Li et al., 2015; Pan et al., 2015; Tang et al., 2015; Du et al., 2016; Liu et al., 2019; Wang et al., 2019). In addition, a biotype was also reported to have evolved multiple resistance to these kinds of herbicide (Li et al., 2017). In contrast to the biotypes described in this research, this reported biotype showed low resistance to the ALS inhibitor mesosulfuron-methyl with an RI of 2.6. Furthermore, this biotype showed high resistance to the ACCase inhibitor fenoxaprop-P-ethyl, with an RI value of 31.2 (Li et al., 2017).

### Target-Site Resistance Mechanisms in This Biotype

Mutations involving the target gene *ALS* and *ACCase* is a common mechanism for ALS and ACCase inhibitor herbicide resistance. In this research, the *ALS* gene in the R biotype was shown to have a Pro-197-Ser mutation both by the SNPs and PCR analyses, this indicate the mutation may be one of a number of resistance mechanisms. Evolved resistance in weeds has been attributed to substitutions at each of the following eight different amino acids: Ala-122, Pro-197, Ala-205, Asp-376, Arg-377, Trp-574, Ser-653, and Gly-654 (Murphy and Tranel, 2019). Mutation at the site of 197 of the *ALS* gene was the most frequently reported mutation type in weed species, and was first found in *Kochia scoparia* (L.) Schrad (Saari et al., 1990). For *B. syzigachne*, only one kind of mutation (Pro-197-Ser) was found among the biotypes resistant to ALS inhibitor (Li et al., 2015). To date, seven kinds of amino acid substitutions in the CT domain of ACCase have been reported as being related to resistance to ACCase inhibitor herbicides in grassy weeds, and six substitutions have been found in *B. syzigachne*. In this research, no mutations were found in the R biotype, this indicated NTSR may be evolved in this biotype.

### Common Metabolic Genes Relative to Multiple Resistance

Herbicide metabolism in weeds is a complex process involving metabolic enzymes (P450, GST, esterase, transporter, oxidase) and regulatory genes (transcription factors, protein kinases, and micro-RNAs) (Kreuz et al., 1996; Van Eerd et al., 2003; Yuan et al., 2007). *Beckmannia syzigachne* has evolved resistance to ACCase inhibitors, and many genes involved in NTSR were discovered using the RNA-seq technology (Pan et al., 2016a). Metabolic genes including CytP450, GST, esterases, peroxidases, ABC transporter B family members, and UDP-glycosyltransferases were identified (Pan et al., 2016a) Esterases break down herbicide molecules, and ABC transporters enhance resistance to herbicides by transporting isolated herbicides and acting as metabolites (Délye, 2013). In this research, one gene was annotated as an ABC transporter G family member, and one gene was annotated as UDP-glycosyltransferase 88F5 may be associated with resistance. Furthermore, two genes (*GST-T3* and *GST-U6*) were found to be overexpressed at different times after mesosulfuron-methly and fenoxaprop-p-ethyl treatment in resistant *B. syzigachne*. What is more, the GST activity in resistant plants higher than the susceptible ones at all the detected times. This result is similar to that reported in a previous study (Pan et al., 2016b). This confirmed that these two genes are related to multiple resistance in this weed.

### Novel Genes Related to Multiple Resistance

In addition to the reported resistance factors in *B. syzigachne*, some new genes related to resistance were also found in this research. Four genes annotated as encoding disease resistance proteins (one for *At1g15890*, one for *At1g58602*, and two for *RPP13*) in the resistant population were expressed at 65.8-1124.0 times higher levels than that in the sensitive biotype. This phenomenon was found both before and after herbicide treatment. This suggested that the disease-resistance protein might be related to the resistance. The expression levels of oxidase (L-pipecolate oxidase and iron/ascorbate family oxidoreductases) in R biotypes were 10.0–103.4 times higher than those in sensitive biotypes after treatment, which may be related to fenoxaprop-p-ethyl metabolism in individuals.

In this study, besides disease-resistant proteins, transcription factors and other regulatory genes were also screened. Protein kinases play key roles in signal transduction pathways, bringing external signals into cells, amplifying cascades of reactions, and passing them to transcription factor proteins to regulate the expression of downstream functional genes. Plant immune receptor is often classified as threonine kinase, and studies have shown that tyrosine phosphorylation in plants has an important regulatory role in innate immunity (Liu et al., 2018). MYC2 has a regulatory role in plant antioxidant capacity (Dombrecht et al., 2007). In this study, MYC3 transcription factors and protein kinase levels in WC11-48 expression were 22.8-917.2 times higher in the resistant biotype than that in the sensitive biotype, but the present study has not reported anything about the relationship between resistance genes and weeds. The roles of these genes in weed resistance mechanisms still need further research.

## Conclusion

In this research, we found a *B. syzigachne* biotype that had evolved multiple resistance to ALS and ACCase inhibitor herbicides, and which showed high resistance to ALS inhibitor and relatively lower resistance to the ACCase ones. A Pro-197-Ser mutation was found in the *ALS* gene and no mutations were found in the *ACCase* gene. Four genes encoding disease resistance proteins, the MYC3 transcription factor, and one gene for blight resistance protein were found related to confer multiple resistance. Of the novel resistance factors, two GST genes (*GST-T3* and *GST-U6*) showed high correlation to this multiple resistance. The results of this research could provide the important resistant factors about multiple resistance to ACCase and ALS-inhibiting herbicide in *B. syzigachne*.

## Data Availability Statement

The raw Illumina sequence reads have been deposited in the NCBI Sequence Read Archive (SRA) database. The accession numbers for the samples with herbicide treatment were SRR10737896 and SRR10737895. And the accession numbers for the samples without herbicide treatment were SRR10742660 and SRR10742659.

## Author Contributions

This research was designed by HC. JW and JC performed the experimental work and the data analysis. XL, HC, DL, ZL, and JC provided helpful suggestions for the data analysis and manuscript revision. JC wrote the manuscript. All authors contributed to the article and approved the submitted version.

## Funding

This research was funded by The National Natural Science Foundation of China (no. 31371952).

## Conflict of Interest

The authors declare that the research was conducted in the absence of any commercial or financial relationships that could be construed as a potential conflict of interest.
