# Supplementary material for: Pro-197-Ser Mutation in ALS and High-Level GST Activities: Multiple Resistance to ALS and ACCase Inhibitors in Beckmannia syzigachne
Source: Front Plant Sci. 2020 Sep 30;11:572610. doi: 10.3389/fpls.2020.572610 (PMC7556300; doi:10.3389/fpls.2020.572610)
Supplement: Supplementary file 1 [file Table_1.docx]

**Supplementary Table S1.** The information of the selected herbicide and the dose design for the bioassay.

| Herbicides classification | Herbicide | Formulatio ^a^ | Manufacturer | Biotypes ^b^ | Dose (g a.i. ha^-1^) ^c^ |
| --- | --- | --- | --- | --- | --- |
| Aryloxyphenoxypropionates (APPs) | fenoxaprop-P-ethyl | 69 g/L EW | Bayer | R | 0, **60**, 120, 240, 480, 960, 1920 |
|  |  |  |  | S | 0, 15, 30, **60**, 120, 240, 480 |
|  | clodinafop-propargyl | 15% WP | Syngenta | R | 0, 12.5, 25, **50**, 100, 200, 400, 800 |
|  |  |  |  | S | 0, 6.25, 12.5, 25, **50**, 100, 200 |
| Cyclohexanediones (CHDs) | clethodim | 240 g/L EC | Cynda Chemical | R | 0, 5, 10, 20, 40, **80**, 160 |
|  |  |  |  | S | 0, 2.5, 5, 10, 20, 40, **80** |
| Phenylpyrazoline (PPZs) | pinoxaden | 5% EC | Syngenta | R | 0, 25, **50**, 100, 200, 400 |
|  |  |  |  | S | 0, 6.25, 12.5, 25, **50**, 100 |

^a^ EC: emulsifiable concentrate; EW: emulsionoil water; WP: wettable powder. ^b^ S, susceptible biotype; R, the resistant biotype. ^c^ The number in bold represents the recommend rate.
