# Supplementary material for: Pro-197-Ser Mutation in ALS and High-Level GST Activities: Multiple Resistance to ALS and ACCase Inhibitors in Beckmannia syzigachne
Source: Front Plant Sci. 2020 Sep 30;11:572610. doi: 10.3389/fpls.2020.572610 (PMC7556300; doi:10.3389/fpls.2020.572610)
Supplement: Supplementary file 2 [file Table_2.docx]

**Table S2.** Summary of *B. syzigachne* transcriptome sequencing, assembly and annotation using different databases.

| Parameters | Reference transcriptome | |
| --- | --- | --- |
| Total raw reads | | 449,583,465 |
| Total clean reads | | 436,188,739 |
| Assembled unigenes | | 118,111 |
| Maximum length | | 21,671 |
| Minimum length | | 201 |
| N50 contig size | | 1,328 |
| N90 contig size | | 563 |
| Annotation in NR (NCBI non-redundant protein sequences) | | 48,600 |
| Annotation in NT (NCBI non-redundant nucleotide sequences) | | 39,869 |
| Annotation in KO (KEGG Ortholog) | | 15,564 |
| Annotation in SwissProt (A manually annotated and reviewed protein sequence database) | | 31,291 |
| Annotation in PFAM (Protein family) | | 39,310 |
| Annotation in GO (Gene Ontology) | | 35,616 |
| Annotation in KOG (Clusters of Orthologous Groups of proteins) | | 25,598 |
| Annotation in KEGG (Kyoto Encyclopedia of Genes and Genomes) | | 7,893 |
| Annotated in at least one database | | 60,108 |
| Annotated in all databases | | 8,179 |
