# Supplementary material for: Pro-197-Ser Mutation in ALS and High-Level GST Activities: Multiple Resistance to ALS and ACCase Inhibitors in Beckmannia syzigachne
Source: Front Plant Sci. 2020 Sep 30;11:572610. doi: 10.3389/fpls.2020.572610 (PMC7556300; doi:10.3389/fpls.2020.572610)
Supplement: Supplementary file 3 [file Table_3.docx]

**Supplementary Table S3**. The designed primers for the target and reference genes from *B. syzigachne* biotypes

| Gene | Foward primer (5´-3´) | Reverse primer (5´-3) |
| --- | --- | --- |
| *UBQ* | CAAGAAGAAGACGTACACCAAG | GACCTTGTAGAACTGGAGGAG |
| *GADPH* | AGGTTATCAATGACAAGTTTGG | ATCAACAGTCTTCTGGGTAGC |
| *CAP* | AAGCCCCAATCAAAATCAACACGAA | AAGAACACCAAGACCCCCTGC |
| c59327_g | CAGCCGTCTCGCACCAAATCG | GCAAGTGGATTTACCGAGGGA |
| c9871_g1 | AAAATAATGGCGGGTGAAGGGGA | GGTTGGACTTGAGGAGGAGGC |
| c81158_g1 | CGGCTGACCCCTATGAACG | GCACCCTCTAGCGTCTCC |
| c61256_g2 | CGAACTGGATGCTTAAATAGAGGGA | GCAGATGGAAGGAAAGAACCCC |
| c52253_g1 | TATCTGTTGTCTGCCCTTCTCA | TAGTGGATGCGTGGGTTCA |
| c30288_g1 | CACACAACCAGAGATGCGGAC | AGTATGGGGTGTCGTTTAGTCTC |
| c64517_g5 | CGGGCACAAACTATCAGGTCATC | CTGCTTCTGACTCTGGGTTTCG |
| C61823_g3 | GCTTCCATGACTTCCTCGACT | CCCTGCTAATCACACGCTCT |
| c50888_g2 | AGCCCCACGGAACCCTAAAAC | AGGTCGTCGGCATCATCAAGA |
| c61143_g4 | ACTCCCCAATCGCTGTCC | CGTAATCACCTACATGGCACAC |
| c71182_g1 | GCAGAGGAGAGAAGCGAAGGAG | GACACCTTTAGCATCATCTCCCC |
| c41866_g1 | CGAAAGGCAGATATACAAGAAAGGG | CGTGAAGAAACAACCAGAACCAG |
| c62003_g3 | TCCTTCTTCAAACACCACCTCT | GGTTCTGTTTCCCTCCGACG |
| c63887_g5 | GGAGTGATTGGCGTTTGGGGA | CCCTGACCCAAGCACGACAT |
| c64184_g2 | TCCTTGCTTCGTCAGTCATTTCAAAC | TTGGAAGAGAGTGTGAGAAATGTGC |
| c51431_g2 | GGAGGCTTCGGACATGGCTTT | TATCTCAACCTCCGCACCCTT |
| c59149_g1 | CCCATTTGCCGTCACAGTCAG | CACACGAAGCAAACCACAAGA |
| c45597_g1 | TCATAGCCTTCCGTTGAGTGTCG | TTATTAAAGGGTGGCTTGGGTTTCA |
| c96180_g1 | AGCAGAGGAAGAAGAAGGTGAAGC | TCACCACCAGCACCTTCTCG |
