# Supplementary material for: Pro-197-Ser Mutation in ALS and High-Level GST Activities: Multiple Resistance to ALS and ACCase Inhibitors in Beckmannia syzigachne
Source: Front Plant Sci. 2020 Sep 30;11:572610. doi: 10.3389/fpls.2020.572610 (PMC7556300; doi:10.3389/fpls.2020.572610)
Supplement: Supplementary file 4 [file Table_4.docx]

**Supplementary Table S4.** GO classification of the annotated unigene in *B. syzigachne*.

| **GO_Term (Level1)** | **GO_Term (Level2)** | **Gene_Number** |
| --- | --- | --- |
| **Cellular Component** | macromolecular complex | 3854 |
|  | cell part | 26589 |
|  | extracellular region | 305 |
|  | symplast | 457 |
|  | cell junction | 457 |
|  | organelle part | 4700 |
|  | membrane-enclosed lumen | 1263 |
|  | virion part | 118 |
|  | cell | 26622 |
|  | virion | 118 |
|  | organelle | 18412 |
|  | extracellular region part | 109 |
| **Molecular Function** | transporter activity | 1777 |
|  | metallochaperone activity | 3 |
|  | enzyme regulator activity | 475 |
|  | nucleic acid binding transcription factor activity | 736 |
|  | structural molecule activity | 821 |
|  | receptor activity | 89 |
|  | molecular transducer activity | 267 |
|  | catalytic activity | 16901 |
|  | binding | 17540 |
|  | antioxidant activity | 361 |
|  | protein binding transcription factor activity | 141 |
| **Biological Process** | biological regulation | 5288 |
|  | signaling | 1338 |
|  | multicellular organismal process | 1208 |
|  | establishment of localization | 3474 |
|  | pigmentation | 2 |
|  | localization | 3584 |
|  | growth | 199 |
|  | negative regulation of biological process | 506 |
|  | positive regulation of biological process | 427 |
|  | reproduction | 802 |
|  | cell proliferation | 22 |
|  | multi-organism process | 410 |
|  | locomotion | 16 |
|  | reproductive process | 768 |
|  | cellular process | 19819 |
|  | metabolic process | 19579 |
|  | cellular component organization or biogenesis | 3336 |
|  | biological adhesion | 7 |
|  | immune system process | 114 |
|  | regulation of biological process | 4659 |
|  | viral reproduction | 16 |
|  | developmental process | 1193 |
|  | response to stimulus | 3773 |
|  | cell killing | 7 |
|  | rhythmic process | 33 |
|  | death | 111 |
|  | nitrogen utilization | 3 |
