# Supplementary material for: Pro-197-Ser Mutation in ALS and High-Level GST Activities: Multiple Resistance to ALS and ACCase Inhibitors in Beckmannia syzigachne
Source: Front Plant Sci. 2020 Sep 30;11:572610. doi: 10.3389/fpls.2020.572610 (PMC7556300; doi:10.3389/fpls.2020.572610)
Supplement: Supplementary file 5 [file Table_5.docx]

**Supplementary Table S5.** KOG classification of putative proteins corresponding to *B. syzigachne* unigenes.

| **KOG_description** | **number** |
| --- | --- |
| RNA processing and modification | 941 |
| Chromatin structure and dynamics | 335 |
| Energy production and conversion | 1230 |
| Cell cycle control, cell division, chromosome partitioning | 695 |
| Amino acid transport and metabolism | 1035 |
| Nucleotide transport and metabolism | 258 |
| Carbohydrate transport and metabolism | 1486 |
| Coenzyme transport and metabolism | 217 |
| Lipid transport and metabolism | 1004 |
| Translation, ribosomal structure and biogenesis | 1290 |
| Transcription | 1320 |
| Replication, recombination and repair | 616 |
| Cell wall/membrane/envelope biogenesis | 303 |
| Cell motility | 13 |
| Posttranslational modification, protein turnover, chaperones | 2881 |
| Inorganic ion transport and metabolism | 704 |
| Secondary metabolites biosynthesis, transport and catabolism | 1174 |
| General function prediction only | 6874 |
| Function unknown | 1066 |
| Signal transduction mechanisms | 2776 |
| Intracellular trafficking, secretion, and vesicular transport | 1136 |
| Defense mechanisms | 278 |
| Extracellular structures | 96 |
| Unamed protein | 2 |
| Nuclear structure | 90 |
| Cytoskeleton | 574 |
