# Supplementary material for: Pro-197-Ser Mutation in ALS and High-Level GST Activities: Multiple Resistance to ALS and ACCase Inhibitors in Beckmannia syzigachne
Source: Front Plant Sci. 2020 Sep 30;11:572610. doi: 10.3389/fpls.2020.572610 (PMC7556300; doi:10.3389/fpls.2020.572610)
Supplement: Supplementary file 6 [file Table_6.docx]

**Supplementary Table S6.** KEGG classification of *B. syzigachne* unigenes.

| Group | Pathway | Gene Number |
| --- | --- | --- |
| Cellular Processes | Transport and catabolism | 318 |
| Cellular Processes | Cell motility | 29 |
| Cellular Processes | Cellular community | 30 |
| Cellular Processes | Cell growth and death | 171 |
| Environmental Information Processiong | Signaling molecules and interaction | 13 |
| Environmental Information Processiong | Membrane transport | 59 |
| Environmental Information Processiong | Signal transduction | 555 |
| Genetic Information Processing | Folding, sorting and degradation | 626 |
| Genetic Information Processing | Transcripition | 327 |
| Genetic Information Processing | Replication and repair | 219 |
| Genetic Information Processing | Translation | 1141 |
| Metabolism | Gobal and overview maps | 758 |
| Metabolism | Metabolism of cofactors and vitamins | 241 |
| Metabolism | Metabolism of terpenoids and polyketides | 280 |
| Metabolism | Nucleotide metabolism | 108 |
| Metabolism | Carbohydrate metabolism | 592 |
| Metabolism | Metabolism of other amino acids | 116 |
| Metabolism | Energy metabolism | 313 |
| Metabolism | Amino acid metabolism | 219 |
| Metabolism | Lipid metabolism | 356 |
| Metabolism | Biosynthesis of other secondary metabolites | 425 |
| Metabolism | Glycan biosynthesis and metabolism | 128 |
| Metabolism | Xenobiotics biodegradation and metabolism | 53 |
| Organismal Systems | Nervous system | 62 |
| Organismal Systems | Digestive system | 112 |
| Organismal Systems | Immune system | 47 |
| Organismal Systems | Excretory system | 32 |
| Organismal Systems | Endocrine system | 213 |
| Organismal Systems | Environmental adaptation | 321 |
| Organismal Systems | sensory system | 1 |
| Organismal Systems | Development | 28 |
