# Supplementary material for: Pro-197-Ser Mutation in ALS and High-Level GST Activities: Multiple Resistance to ALS and ACCase Inhibitors in Beckmannia syzigachne
Source: Front Plant Sci. 2020 Sep 30;11:572610. doi: 10.3389/fpls.2020.572610 (PMC7556300; doi:10.3389/fpls.2020.572610)
Supplement: Supplementary file 7 [file Table_7.docx]

**Supplementary Table S7.** The SNPs analysis by the RNA-Seq for *ALS* gene (c38811_g1) in different samples.

| **Position** | **Reference ^a^** | **Alter ^b^** | **Samples/reads ^c^** | | | | | |
| --- | --- | --- | --- | --- | --- | --- | --- | --- |
| **629** | **C** | **T** | **CK1004_1** | **CK1004_2** | **CK1004_3** | **CK1148_1** | **CK1148_2** | **CK1148_3** |
|  |  |  | **247,1** | **248,0** | **247,2** | **2,247** | **3,244** | **3,247** |
|  |  |  | **M1004_1** | **M1004_2** | **M1004_3** | **M1148_1** | **M1148_2** | **M1148_3** |
|  |  |  | **248,1** | **250,0** | **248,0** | **2,246** | **0,249** | **2,248** |
|  |  |  | **F1004_1** | **F1004_2** | **F1004_3** | **F1148_1** | **F1148_2** | **F1148_3** |
|  |  |  | **250,0** | **250,0** | **248,1** | **4,246** | **2,245** | **2,247** |
| **820** | **G** | **A** | **CK1004_1** | **CK1004_2** | **CK1004_3** | **CK1148_1** | **CK1148_2** | **CK1148_3** |
|  |  |  | **2,179** | **1,215** | **0,250** | **249,0** | **211,0** | **241,0** |
|  |  |  | **M1004_1** | **M1004_2** | **M1004_3** | **M1148_1** | **M1148_2** | **M1148_3** |
|  |  |  | **0,249** | **2,212** | **1,248** | **250,0** | **246,1** | **249,0** |
|  |  |  | **F1004_1** | **F1004_2** | **F1004_3** | **F1148_1** | **F1148_2** | **F1148_3** |
|  |  |  | **0,250** | **0,247** | **1,248** | **247,2** | **250,0** | **250,0** |

**Note:** ^a^ “Reference” means nucleobase at this position for the *ALS* gene in the reference species; ^b^ “Alter” means the nucleobase at this position for the *ALS* gene in the detected samples in *B. syzigachne*; ^c^ the reads numbers were listed under the sample numbers, and the first number means the reads number of the *ALS* unigene which same to the reference species, the second number means the reads number of *ALS* unigene which was changed.
