# Supplementary material for: Pro-197-Ser Mutation in ALS and High-Level GST Activities: Multiple Resistance to ALS and ACCase Inhibitors in Beckmannia syzigachne
Source: Front Plant Sci. 2020 Sep 30;11:572610. doi: 10.3389/fpls.2020.572610 (PMC7556300; doi:10.3389/fpls.2020.572610)
Supplement: Supplementary file 8 [file Table_8.docx]

**Supplementary Table S8.** Descriptive statistics of three candidate housekeeping genes based on their crossing point (CP)

| Factor ^a^ |  | *UBQ* | *CAP* | *GADPH* |
| --- | --- | --- | --- | --- |
| N |  | 24 | 24 | 24 |
| GM [CP] |  | 22.70 | 25.30 | 21.63 |
| AM [CP] |  | 22.71 | 25.32 | 21.65 |
| Min [CP] |  | 20.97 | 22.82 | 20.04 |
| Max [CP] |  | 24.14 | 27.26 | 22.94 |
| SD [± CP] |  | 0.68 | 0.76 | 0.70 |
| CV [± CP] |  | 3.00 | 3.01 | 3.25 |

^a^ N: the number of samples; GM [CP]: the geometric mean of CP; AM [CP]: the arithmetic mean of CP; Min [CP] and Max [CP]: the extreme values of CP; SD [CP]: the standard deviation of CP; CV [CP]: coefficient of variation expressed as a percentage of the CP level.
